# Hyperbranched Cationic Glycogen Derivative-Mediated I*κ*B*α* Gene Silencing Regulates the Uveoscleral Outflow Pathway in Rats

**DOI:** 10.1155/2020/8206849

**Published:** 2020-12-17

**Authors:** Rui Zeng, Jinmiao Li, Haijun Gong, Jiahao Luo, Zijing Li, Zhaoxing Ou, Si Zhang, Liqun Yang, Yuqing Lan

**Affiliations:** ^1^Department of Ophthalmology, Guangdong Provincial Key Laboratory of Malignant Tumor Epigenetics and Gene Regulation, Sun Yat-sen Memorial Hospital, Sun Yat-sen University, Guangzhou 510120, China; ^2^Department of Polymer and Material Science, School of Chemistry, Key Laboratory for Polymeric Composite and Functional Materials of Ministry of Education, Guangdong Provincial Key Laboratory for High-Performance Polymer-Based Composites, Sun Yat-sen University, Guangzhou 510275, China

## Abstract

The role of the I*κ*B/NF-*κ*B signaling pathway in the uveoscleral outflow pathway was investigated with I*κ*B*α* gene silencing mediated by the 3-(dimethylamino)-1-propylamine-conjugated glycogen (DMAPA-Glyp) derivative. The I*κ*B*α*-siRNA-loaded DMAPA-Glyp complex was transfected into the ciliary muscles of rats by intracameral injection (labeled as the DMAPA-Glyp+siRNA group). The Lipofectamine™ 2000 (Lipo)/siRNA complex and the naked siRNA were set as the controls. The mRNA and protein expression of I*κ*B*α*, NF-*κ*Bp65, and MMP-2 were analyzed by real-time PCR, western blotting, and *in situ* gelatin zymography. Nuclear translocation of NF-*κ*Bp65 was analyzed by immunofluorescence. Rat intraocular pressure (IOP) was monitored pre- and postinjection. Gene transfection efficiency and toxicity of the DMAPA-Glyp derivative were also evaluated. After RNA interference (RNAi), I*κ*B*α* mRNA and protein expression were significantly inhibited. NF-*κ*Bp65 mRNA and protein expression showed no significant differences. Nevertheless, nuclear translocation of NF-*κ*Bp65 occurred in the DMAPA-Glyp+siRNA group. Both mRNA expression and activity of MMP-2 increased, with the largest increase in the DMAPA-Glyp+siRNA group. IOP in the DMAPA-Glyp+siRNA group fell to the lowest level on day 3 after RNAi. The levels of Cy3-siRNA in the ciliary muscle of the DMAPA-Glyp+siRNA group did not significantly decrease over time. At 7 and 14 d after RNAi, no significant pathological damage was detectable in the eyes injected with the DMAPA-Glyp derivative or the DMAPA-Glyp/siRNA complex. Taken together, our results suggest that downregulation of I*κ*B*α* expression in the ciliary muscle plays a crucial role in reducing the IOP values of rats. I*κ*B*α* may become a new molecular target for lowering IOP in glaucoma. The DMAPA-Glyp derivative is safe and feasible as an effective siRNA vector in rat eyes.

## 1. Introduction

Glaucoma is the second leading cause of blindness in the world according to the World Health Organization [1]. Pathological intraocular hypertension is the main risk factor leading to optic nerve damage in glaucoma. Lowering intraocular pressure (IOP) is currently the only method that has been strictly proven to be an effective approach to glaucoma treatment [2]. The IOP-lowering eye drops currently in clinical use must be administered at least once per day and require long-term use, which may damage the ocular surface and cause various ocular symptoms. Consequently, the compliance of patients often declines, leading to irreversible impairment of visual function. Therefore, it is essential to find a means of lowering IOP that offers a better pressure-lowering and more long-lasting effect with fewer side effects.

The vast majority of glaucoma cases result from the elevation of IOP due to an increasing aqueous humor outflow resistance. Uveoscleral drainage of the aqueous humor accounts for 10–20% of the total aqueous humor outflow and is a non-pressure-dependent pathway which is functional when the IOP is higher than 4 mmHg [3] and therefore plays a major role in the treatment of glaucoma. The ciliary muscle is the flow restriction site of this pathway. Remodeling of the ciliary muscle extracellular matrix (ECM) plays a nonnegligible role in the drainage of the aqueous humor via this pathway. An imbalance between matrix metalloproteinases (MMPs) and their endogenous inhibitors, tissue inhibitors of matrix metalloproteinases (TIMPs), is one of the major factors leading to the abnormal deposition of ECM in the aqueous humor outflow pathway [4]. Therefore, MMPs/TIMPs are critical regulators of IOP.

Previous works have reported that prostaglandins could degrade ciliary muscle ECM by promoting the synthesis of MMPs or by increasing MMP activity in the uveoscleral pathway, resulting in reducing aqueous humor outflow resistance, increasing aqueous humor outflow, and lowering IOP [5, 6]. However, the upstream molecular regulation mechanism of such effect is unclear at present. As an important transcription factor discovered recently, nuclear factor kappa B (NF-*κ*B) is expressed in a variety of cells, which constitutes a system together with its inhibitor I*κ*B that participates in various physiological and pathological processes as well as in the regulation of some MMPs [7, 8]. We had recently demonstrated in human ciliary muscle cells *in vitro* that the downregulation of I*κ*B*α* expression by RNA interference (RNAi) could trigger the transcriptional activity of NF-*κ*B, thereby increasing MMP-2 expression and downregulating TIMP-2 expression [9]. However, it still remains unclear whether the I*κ*B/NF-*κ*B system participates in the regulation of MMP-2 expression and activity, thereby affecting the uveoscleral outflow *in vivo*.

The RNAi technique has been used extensively in research on the molecular and biochemical mechanisms of intracellular signaling pathways. Small interfering RNA (siRNA), which is an important effector of RNAi, can be easily degraded by nucleases and is not readily transported across the cellular membrane due to its hydrophilicity and negative charge. Therefore, to prevent siRNA degradation and promote siRNA transfection efficiencies, it is necessary to select an appropriate delivery vector.

Nanoparticle delivery vectors, currently the most effective nonviral vectors, have high transfection efficiencies and offer many advantages such as low immunogenicity, low toxicity, high stability, and ease of preparation [10], yielding great application prospects in gene therapy [11]. In a previous study, we successfully synthesized a hyperbranched cationic glycogen derivative, 3-(dimethylamino)-1-propylamine-conjugated glycogen (DMAPA-Glyp), and proved that DMAPA-Glyp, which exhibits good biocompatibility and low cytotoxicity, improves the stability of siRNA in serum and prolongs the interference effect of siRNA [12].

In the present study, I*κ*B*α*-siRNA was transfected into rat ciliary muscle *in vivo* mediated by DMAPA-Glyp. The resulting changes in I*κ*B*α*, NF-*κ*Bp65, and MMP-2 expression in the ciliary muscle and in the IOP of rats were observed. The objectives of the study were to further investigate the molecular mechanism of the I*κ*B/NF-*κ*B system in regulation of the uveoscleral outflow pathway and to evaluate the efficacy and safety of DMAPA-Glyp as a siRNA vector in rat eyes.

## 2. Materials and Methods

### 2.1. Materials

The DMAPA-Glyp derivative and the solution containing the DMAPA-Glyp/siRNA complex were prepared according to our previous work [12]. 100 mg of DMAPA-Glyp was dissolved in 10 ml of sterilized triple-distilled water and stored at room temperature overnight to formulate a DMAPA-Glyp stock solution at 10 mg/ml. The stock solution was stored at 4°C and diluted to a 1 mg/ml working solution in sterilized triple-distilled water before use. Based on the weight ratio of *W*_DMAPA−Glyp_/*W*_siRNA_ = 5, Cy3-labeled siRNA (1 *μ*g) was added to 5 *μ*l of solution containing 5 *μ*g of DMAPA-Glyp. The mixture was incubated at room temperature for 10–15 min to yield the DMAPA-Glyp/siRNA complex.

The siRNA used in the experiments was designed and synthesized by Guangzhou RiboBio Co., Ltd. (Guangzhou, China). The sequences of the siRNA duplex targeting the rat I*κ*B*α* gene (NM_001105720) were as follows: sense strand, 5′ CUACGAUGACUGUGUGUUUdTdT 3′; antisense strand, 5′ AAACACACAGUCAUCGUAGdTdT 3′. A nonspecific control siRNA duplex (NC-siRNA) and a Cy3- or Cy5-labeled NC-siRNA duplex, all 21 bp in length, were also prepared.

### 2.2. Animals

All animal procedures and methods were conducted in accordance with NIH guidelines for the care and use of laboratory animals and the ARVO Statement for the Use of Animals in Ophthalmic and Vision Research. The research protocol was approved by the Ethics Committee of Zhongshan Ophthalmic Center at Sun Yat-sen University in China.

Male Wistar rats, free of eye disease and weighing 200–250 g, were provided by the Experimental Animal Center of Sun Yat-sen University (Guangzhou, China). The rats were acclimatized in a specific pathogen-free (SPF) laboratory for 1 week before initiation of the study. Rats were anesthetized with an intraperitoneal injection of 10% chloral hydrate (300 mg/kg of body weight) and with topical 0.5% proxymetacaine hydrochloride drops (Alcaine, Alcon, Fort Worth, USA). At the end of the experiments, rats were euthanized by an overdose of 10% chloral hydrate. The animal experiment was conducted in the Experimental Animal Center of Zhongshan Ophthalmic Center.

### 2.3. Selection of Optimal Delivery Route

The rats were randomly divided into three groups: an intravitreal injection group, a ciliary muscle injection group, and an intracameral injection group. The DMAPA-Glyp/Cy3-siRNA complexes were transfected into rat eyes with these three delivery routes, respectively, and the optimal one was then selected. All injections were administered in the left eye of the rats.

#### 2.3.1. Intravitreal Injection

The rats were anesthetized as described above. The superonasal sclera was exposed, and a microsyringe with a 33-gauge needle (Hamilton Bonaduz AG, Switzerland) was used to inject 10 *μ*l of the DMAPA-Glyp/Cy3-siRNA complex (containing 1 *μ*g of siRNA) 1.5 mm behind the corneal limbus into the vitreous cavity under a surgical microscope (Leica, Heerbrugg, Switzerland) [13]. Conventional feeding was used after injection.

#### 2.3.2. Ciliary Muscle Injection

The rats were anesthetized as described above. We carried out the intraciliary muscle injection through a tunneled corneal incision. A microsyringe with a 33-gauge needle was inserted slightly deeper for a distance of 1 mm until it had crossed the corneal limbus. Then, 10 *μ*l of the DMAPA-Glyp/Cy3-siRNA complex (containing 1 *μ*g of siRNA) was injected under a surgical microscope [14]. Conventional feeding was performed after injection.

#### 2.3.3. Intracameral Injection

The rats were anesthetized as described above. A microsyringe (33-gauge needle) was used to inject 10 *μ*l of the DMAPA-Glyp/Cy3-siRNA complex (containing 1 *μ*g of siRNA) through the peripheral cornea into the anterior chamber of rats under a surgical microscope. Care was taken to prevent damage to the lens and iris [15]. Conventional feeding was performed after injection.

#### 2.3.4. Selection of Optimal Delivery Route

The optimal way for effective delivery of the transfection composite to the ciliary muscle was selected. 24 h after injection, rat eyes were removed under anesthesia and embedded in OCT (Sakura Finetek USA Inc., Torrance, USA). After quick freezing at -80°C, 7 *μ*m frozen sections were prepared which were fixed in 4% paraformaldehyde and incubated with 4,6-diamidino-2-phenylindole (DAPI) nuclear staining solution (Beyotime Institute of Biotechnology, Shanghai, China). The distribution of DMAPA-Glyp/Cy3-siRNA complexes (red fluorescence) in rat ciliary muscle was observed under a fluorescence microscope (Carl Zeiss Meditec AG, Jena, Germany).

#### 2.3.5. Immunofluorescence Staining of *α*-SMA

Frozen sections of rat eyes from the intracameral injection group were used for immunofluorescence staining of *α*-smooth muscle actin (*α*-SMA) to localize ciliary muscle. They were fixed in 4% paraformaldehyde for 10 min and blocked with normal goat serum at room temperature for 1 h. This was followed by incubation with anti-*α*-SMA mouse monoclonal antibody (1 : 100) (Sigma-Aldrich, St. Louis, USA) at 4°C overnight and subsequent incubation with fluorescein isothiocyanate- (FITC-) labeled goat anti-mouse IgG (1 : 50) (Sigma-Aldrich, St. Louis, USA) at room temperature for 2 h. Finally, the sections were incubated with DAPI nuclear staining solution at room temperature for 5 min, mounted with an antifluorescent quenching agent (Beyotime Institute of Biotechnology, Shanghai, China) and observed under a fluorescence microscope.

### 2.4. Optimization of siRNA Transfection Dose

#### 2.4.1. Fluorescence Microscopy

The rats were randomly divided into three groups: a 1 *μ*g group, a 3 *μ*g group, and a 5 *μ*g group. Complexes containing various doses of Cy3-siRNA and DMAPA-Glyp were prepared as described above, and 10 *μ*l of each complex was intracamerally injected into rat eyes. 24 h later, the eyes were removed under anesthesia to prepare frozen sections. The distribution of red fluorescence in the ciliary muscles of the rats in each group was observed under a fluorescence microscope.

#### 2.4.2. Semiquantitative RT-PCR

The rats were randomly divided into five groups: a phosphate-buffered saline (PBS) group; an NC-siRNA group; and 1 *μ*g, 3 *μ*g, and 5 *μ*g I*κ*B*α*-siRNA groups. I*κ*B*α*/NC-siRNA and DMAPA-Glyp were formulated into complexes as described above, and 10 *μ*l of a complex was intracamerally injected into rat eyes. The PBS group was injected with an equal volume of sterilized PBS as the control. 24 h later, the eyeballs were removed under anesthesia, and the ciliary body was separated under a dissecting microscope (Carl Zeiss Meditec AG, Jena, Germany) and placed in a RNase-free glass homogenizer tube. 300 *μ*l of RNAiso Plus (Takara Bio Inc., Kyoto, Japan) was added to the sample, and the sample was homogenized in an ice bath until no visible particles remained. The homogenate was transferred to an RNase-free 1.5 ml centrifuge tube, allowed to stand at room temperature for 5 min, and centrifuged at 12,000 rpm and 4°C for 5 min. The supernatant was removed to a new RNase-free centrifuge tube. Total RNA was extracted according to the protocol provided with the RNAiso Plus kit. The cDNA was synthesized by reverse transcription using the PrimeScript™ RT Reagent Kit (Takara Bio Inc., Kyoto, Japan) according to the manufacturer's protocol. Semiquantitative RT-PCR was performed using a Premix Ex Taq™ Version 2.0 (loading dye mix) kit (Takara Bio Inc., Kyoto, Japan). The cycle parameters were as follows: 98°C for 10 sec, 60°C for 30 sec, and 72°C for 30 sec, with 24 cycles for glyceraldehyde 3-phosphate dehydrogenase (GAPDH) and 30 cycles for I*κ*B*α*. The primer sequences of GAPDH and I*κ*B*α* are listed in Table 1. The PCR products were subjected to electrophoresis on 2% agarose gels and visualized under ultraviolet illumination using an INFINITY 3026 gel image machine (Vilber Lourmat Deutschland GmbH, Eberhardzell, Germany).

### 2.5. Evaluation of the siRNA Transfection Efficiency of the DMAPA-Glyp Derivative

The rats were randomly divided into three groups: the siRNA group, the Lipofectamine™ 2000 (Lipo) (Invitrogen, Carlsbad, USA) +siRNA group, and the DMAPA-Glyp+siRNA group. DMAPA-Glyp and Lipo were used separately to load 5 *μ*g of Cy3-siRNA, and 10 *μ*l of each complex was intracamerally injected into rat eyes, with naked siRNA as the control. At 24, 48, and 72 h after injection, the eyes were removed under anesthesia, and frozen sections were prepared. The distribution of red fluorescence in the ciliary muscle of rats in each group was observed under a fluorescence microscope.

### 2.6. The DMAPA-Glyp Derivative-Mediated I*κ*B*α*-siRNA Transfection In Vivo

The experiment involved six groups of rats (Table 2). Each group was transfected by intracameral injection as described above with a volume of 10 *μ*l per eye.

#### 2.6.1. I*κ*B*α*, NF-*κ*Bp65, and MMP-2 Gene Expression Assay after RNAi

*(1) Real-Time PCR*. The rats were anesthetized at 24, 48, and 72 h after intracameral injection. The eyes were removed, and the ciliary body was separated on ice. Total RNA was extracted for real-time PCR to measure I*κ*B*α*, NF-*κ*Bp65, and MMP-2 mRNA expression at various time points after RNAi. Total RNA extraction and reverse transcription were conducted as described above. PCR amplification was performed on a LightCycler 480 real-time fluorescence quantitative PCR machine using the SYBR® Premix Ex Taq™ kit (Takara Bio Inc., Kyoto, Japan). The PCR amplification program was as follows: predenaturation (1 cycle, 95°C for 30 sec), PCR reaction (40 cycles, 95°C for 5 sec and 60°C for 30 sec), melting curve analysis (1 cycle, 95°C for 5 sec, 60°C for 1 min, and 95°C), and cooling (1 cycle, 50°C for 30 sec). The relative expression of target gene mRNA was calculated and analyzed by the 2^−ΔΔCt^ method [16]. The primer sequences of GAPDH, I*κ*B*α*, NF-*κ*Bp65, and MMP-2 are listed in Table 1.

*(2) Western Blotting*. Total protein was extracted from the ciliary bodies to measure I*κ*B*α* and NF-*κ*Bp65 protein expression at 24, 48, and 72 h after RNAi by western blot assays. 20 *μ*g of protein was loaded in each lane of SDS-polyacrylamide gel, and the expression of *β*-actin was detected as a loading control. The protein was separated by electrophoresis on SDS-polyacrylamide gels and transferred to a polyvinylidene difluoride membrane. The membrane was incubated in a 5% BSA blocking solution at room temperature for 1 h, followed by overnight incubation at 4°C in anti-I*κ*B*α* mouse monoclonal antibody (1 : 1000), anti-NF-*κ*Bp65 rabbit monoclonal antibody (1 : 1000), or anti-*β*-actin mouse monoclonal antibody (1 : 2000) (Cell Signaling Technology, Danvers, USA). The membrane was then placed in goat anti-mouse or goat anti-rabbit IgG conjugated with horseradish peroxidase (1 : 10,000) (Abcam, Cambridge, USA) and incubated at room temperature for 1 h. The bands were visualized by an enhanced chemiluminescence kit (Thermo Fisher Scientific, Waltham, USA). The grayscale of the bands was scanned using ImageJ software. The grayscale ratio of the target protein and *β*-actin was used to indicate the relative expression of the target protein.

#### 2.6.2. NF-*κ*Bp65 Nuclear Translocation and MMP-2 Activity Assays after RNAi

At 24, 48, and 72 h after intracameral injection, the rat eyes were immediately removed under anesthesia and embedded in OCT. After quick freezing at -80°C, 7 *μ*m thick and 10 *μ*m thick frozen sections were prepared. The former were used for immunofluorescence staining of NF-*κ*Bp65, as described above, at an anti-NF-*κ*Bp65 rabbit monoclonal antibody dilution of 1 : 50 (Cell Signaling Technology, Danvers, USA). The sections were observed under a fluorescence microscope. Assessment of the NF-*κ*Bp65 nuclear translocation was carried out by quantifying the intensity of green fluorescence in the nuclei of ciliary muscle area in three sections for each eye by using Image-Pro Plus software (Media Cybernetics, US).

The 10 *μ*m thick frozen sections were used for *in situ* gelatin zymography to analyze MMP-2 activity in rat ciliary muscle, which was assayed using an *in situ* gelatin zymography fluorescence staining kit for MMP-2 (GenMed Scientifics Inc., Wilmington, USA) with the preparation kept away from light. The staining solution (Reagent B) was thawed and preheated at room temperature, and the colloidal solution (Reagent A) was heated and thawed in a constant-temperature water bath at 60°C. Then, 80 *μ*l of the colloidal solution was pipetted into a 1.5 ml centrifuge tube and incubated in a temperature-controlled water bath at 37°C for 10 min. Meanwhile, the staining solution was instantaneously preheated at 37°C, and 10 *μ*l of the preheated staining solution was added to the colloidal solution. The mixture was immediately added to unfixed frozen sections, and the sections were incubated at 4°C for 10 min until the colloid had coagulated. The sections were then incubated at 37°C for 24 h and observed under a fluorescence microscope. The fluorescence level counting in the area of the ciliary muscle was performed to measure the MMP-2 activity by using Image-Pro Plus software.

#### 2.6.3. Analysis of IOP Changes in Rats after RNAi

The rats were randomly divided into the following four groups: the PBS group, the DMAPA-Glyp group, the DMAPA-Glyp+NC group, and the DMAPA-Glyp+siRNA group. We measured rat IOP under topical anesthesia continuously for 3 d preinjection to determine the baseline value. And then, IOP was measured at 1–5 d, 1 w, and 2 w after intracameral injection by the same person at the same time of day (14:00) using a Tono-Pen XL Applanation Tonometer (Reichert, NY, USA). Each eye was measured three times, and the mean of the three measurements was taken as the IOP of the eye.

#### 2.6.4. Evaluation of the Toxicity of the DMAPA-Glyp Derivative

At 7 d and 14 d after intracameral injection, anterior segment photography was conducted under a slit-lamp microscope (Carl Zeiss Meditec AG, Jena, Germany) to observe the occurrence of cataract, corneal edema, iris hyperemia, and hemorrhage among the rats in Section 2.6.3. Besides, the eyeballs of each group were removed at 7 d and 14 d after RNAi, and paraffin sections were stained with hematoxylin-eosin (HE). The rat eyes were fixed in 4% paraformaldehyde at 4°C for 24 h. After dehydration in a graded alcohol series and paraffin embedding, 4 *μ*m sections were prepared. The sections were dewaxed with xylene followed by gradient hydration. All sections were stained with hematoxylin (Beyotime Institute of Biotechnology, Shanghai, China) for 5 min and rinsed with tap water. The sections were then differentiated with HCl-ethanol for 10 sec, immersed in tap water for 15 min, and placed in an eosin solution (Beyotime Institute of Biotechnology, Shanghai, China) for 1 min. Finally, the sections were conventionally dehydrated, cleared in xylene, and mounted in neutral balsam and then observed on a microscope (Leica, Heerbrugg, Switzerland).

### 2.7. Statistical Analysis

The data were analyzed using SPSS 13.0 (SPSS Inc., Chicago, USA). The experimental data that were normally distributed are expressed as the mean ± standard deviation (−*χ* ± *s*). Multiple comparisons of the means were conducted using one-way ANOVA. Pairwise comparison was performed using the LSD *t*-test. Comparisons of mean values between two groups were analyzed by Student's *t*-test. All experiments were repeated at least three times under the same conditions, and the final results were averaged. The test of significance was conducted at the level *α*=0.05.

## 3. Results

### 3.1. Selection of Delivery Route

**Figure 1:**
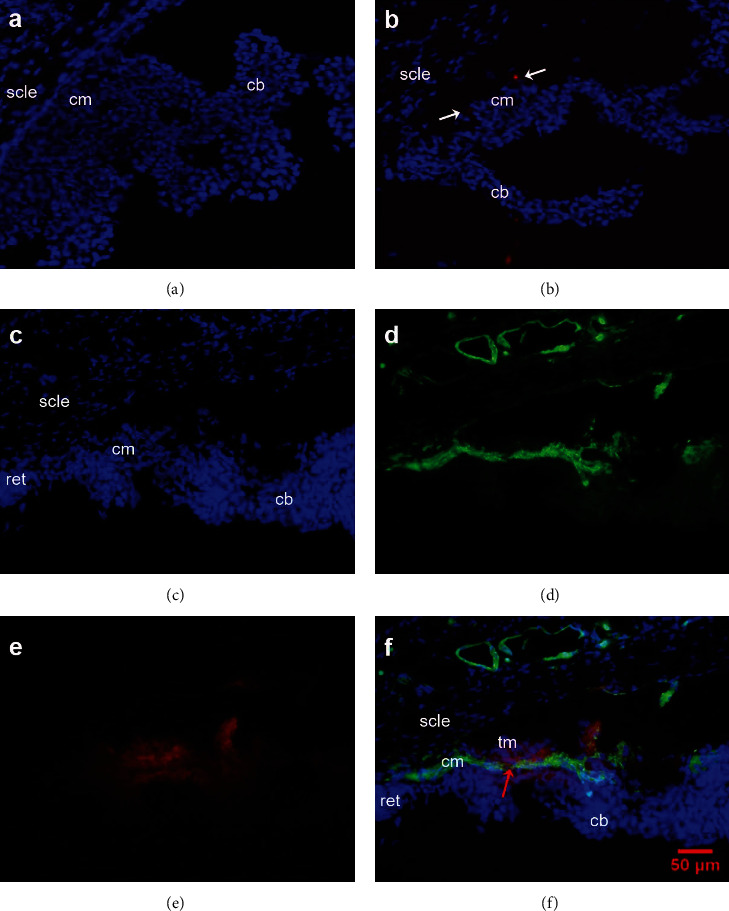
The distribution of DMAPA-Glyp/Cy3-siRNA complexes in rat ciliary muscle after transfection via three different delivery routes (×200) (*n* = 6 per group). *Notes*. (a) Intravitreal injection group. (b) Ciliary muscle injection group—the white arrows indicate scattered fluorescence (red) in the ciliary muscle. (c–f) Intracameral injection group: (c) nuclei; (d) ciliary muscle fibers—*α*-SMA-positive immunofluorescence staining (green); (e) DMAPA-Glyp/Cy3-siRNA complexes (red); (f) merging of (c), (d), and (e)—the red arrow indicates DMAPA-Glyp/Cy3-siRNA complexes in the ciliary muscle. Abbreviations: cb—ciliary body; cm—ciliary muscle; ret—retina; scle—sclera; tm—trabecular meshwork; DMAPA-Glyp—3-(dimethylamino)-1-propylamine-conjugated glycogen; *α*-SMA—*α*-smooth muscle actin.

There was no remarkable distribution of red fluorescence in the ciliary body after intravitreal injection (Figure 1(a)); only a small amount of scattered fluorescence was visible in the ciliary muscle and ciliary processes after ciliary body injection (Figure 1(b)). A large amount of the DMAPA-Glyp/Cy3-siRNA complexes were found in the ciliary muscle after intracameral injection which was localized by *α*-SMA-positive immunofluorescence staining (green fluorescence). Some of them were observed in the trabecular meshwork as well. Meanwhile, the complexes were only rarely observed in the iris and corneal endothelium after intracameral injection (Figures 1(c)–1(f)).

### 3.2. Optimization of siRNA Transfection Dose

**Figure 2:**
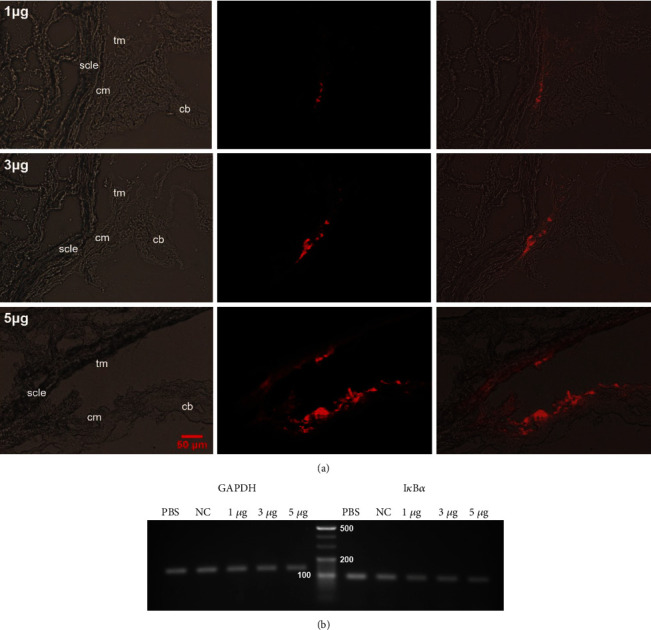
Optimization of siRNA transfection dose. *Notes*. (a) The distribution of various doses of Cy3-siRNA (red) in the rat ciliary muscle (×200) (*n* = 6 per group). (b) Agarose gel electrophoresis of semiquantitative RT-PCR analysis at 24 h after transfection of different doses of I*κ*B*α*-siRNA (*n* = 6 per group). Abbreviations: cb—ciliary body; cm—ciliary muscle; scle—sclera; tm—trabecular meshwork; GAPDH—glyceraldehyde 3-phosphate dehydrogenase.

The group of rats that received 5 *μ*g of I*κ*B*α*-siRNA showed the strongest fluorescence distribution in the rat ciliary muscle and the greatest inhibitory effect on I*κ*B*α* mRNA in the ciliary muscle at 24 h among rats transfected with various doses of siRNA (Figure 2).

### 3.3. Evaluation of the siRNA Transfection Efficiency of the DMAPA-Glyp Derivative

**Figure 3:**
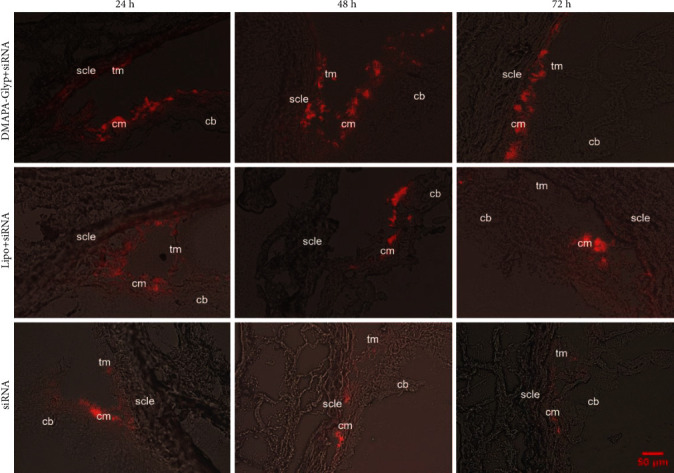
The distribution of Cy3-siRNA in the ciliary muscle at 24, 48, and 72 h after intracameral injection (×200) (*n* = 4 per group). Abbreviations: cb—ciliary body; cm—ciliary muscle; scle—sclera; tm—trabecular meshwork; DMAPA-Glyp—3-(dimethylamino)-1-propylamine-conjugated glycogen; Lipo—Lipofectamine™ 2000.

Fluorescence microscopic observation showed that the distribution of Cy3-siRNA in the ciliary muscle decreased over time in the Lipo+siRNA and siRNA groups. However, there was no remarkable decrease in Cy3-siRNA in the DMAPA-Glyp+siRNA group. A large amount of red fluorescence was distributed in the ciliary muscle of rats in that group at 72 h after transfection. Comparison of different groups at the same time points revealed higher fluorescence distribution in the ciliary muscle in the DMAPA-Glyp+siRNA group than in the other two groups (Figure 3).

### 3.4. Changes of I*κ*B*α* Gene Expression after RNAi

**Figure 4:**
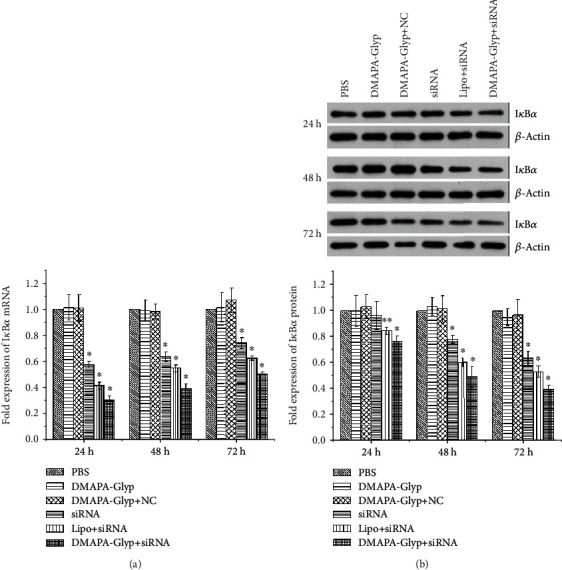
Examination of I*κ*B*α* mRNA and protein levels at 24, 48, and 72 h after RNAi. The error bars represent the standard deviation calculated from three parallel experiments (*n* = 6 per group). *Notes*. (a) I*κ*B*α* mRNA expression was quantified by real-time PCR. Expression levels were normalized with GAPDH. (b) I*κ*B*α* protein expression was examined by western blot. The I*κ*B*α* and *β*-actin bands were analyzed by densitometry, and the values were normalized with *β*-actin, which were represented in the bar graph. ^∗^*P* < 0.01 and ^∗∗^*P* < 0.05 compared with the PBS group, the DMAPA-Glyp group, and the DMAPA-Glyp+NC group. Abbreviations: PBS—phosphate-buffered saline; DMAPA-Glyp—3-(dimethylamino)-1-propylamine-conjugated glycogen; NC—nonspecific control; Lipo—Lipofectamine™ 2000.

I*κ*B*α* mRNA expression was significantly decreased in the three I*κ*B*α* siRNA-transfected groups (the siRNA group, the DMAPA-Glyp+siRNA group, and the Lipo+siRNA group) compared with the three control groups (the PBS group, the DMAPA-Glyp group, and the DMAPA-Glyp+NC group) at various time points after intracameral injection (24 h: *F* = 179.339, *df* = 35, *P* < 0.01; 48 h: *F* = 190.548, *df* = 35, *P* < 0.01; and 72 h: *F* = 85.191, *df* = 35, *P* < 0.01). The lowest I*κ*B*α* mRNA expression occurred 24 h after transfection, and the inhibition rate gradually declined with time. There was a stronger inhibitory effect on mRNA in the DMAPA-Glyp+siRNA group than in the Lipo+siRNA and siRNA groups; the levels of I*κ*B*α* mRNA in the DMAPA-Glyp+siRNA group showed 69.3%, 61.1%, and 49.9% inhibition at 24, 48, and 72 h, respectively, after transfection (Figure 4(a)).

At the level of protein expression, I*κ*B*α* protein expression gradually decreased over time in the three I*κ*B*α* siRNA-transfected groups. The decrease in I*κ*B*α* protein expression was statistically significant in all three I*κ*B*α*-siRNA-transfected groups at most time points, except that there was no significant difference in I*κ*B*α* protein expression in the siRNA group compared with the three control groups at 24 h after transfection (24 h: *F* = 10.674, *df* = 35, *P* < 0.01; 48 h: *F* = 85.078, *df* = 35, *P* < 0.01; and 72 h: *F* = 98.423, *df* = 35, *P* < 0.01). The DMAPA-Glyp+siRNA group showed a more remarkable gene silencing effect than the Lipo+siRNA and siRNA groups, with inhibition of 23.3%, 51.0%, and 61.0% at 24, 48, and 72 h, respectively, after transfection (Figure 4(b)).

### 3.5. Changes in NF-*κ*Bp65 Gene Expression after RNAi

**Figure 5:**
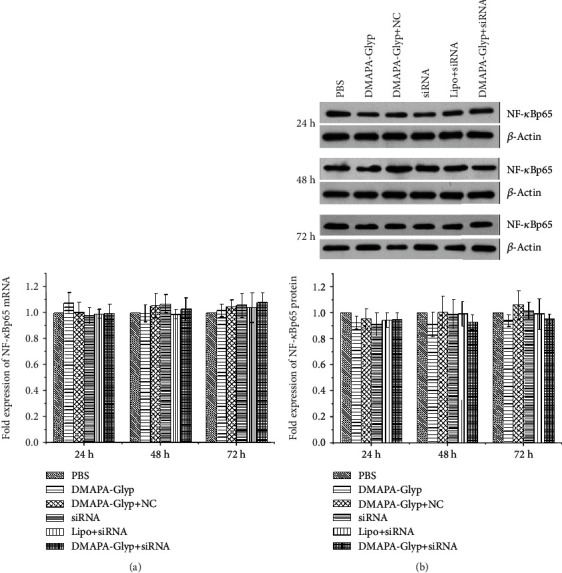
Examination of NF-*κ*Bp65 mRNA and protein levels at 24, 48, and 72 h after RNAi. The error bars represent the standard deviation calculated from three parallel experiments (*n* = 6 per group). *Notes*. (a) NF-*κ*Bp65 mRNA expression was quantified by real-time PCR. Expression levels were normalized with GAPDH. (b) NF-*κ*Bp65 protein expression was examined by western blot. The NF-*κ*Bp65 and *β*-actin bands were analyzed by densitometry, and the values were normalized with *β*-actin, which were represented in the bar graph. Abbreviations: PBS—phosphate-buffered saline; DMAPA-Glyp—3-(dimethylamino)-1-propylamine-conjugated glycogen; NC—nonspecific control; Lipo—Lipofectamine™ 2000.

**Figure 6:**
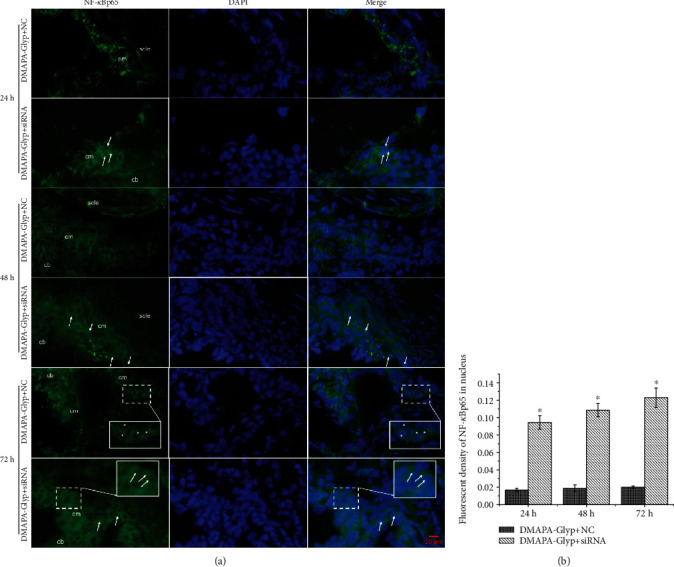
NF-*κ*Bp65 nuclear translocation at 24, 48, and 72 h after RNAi by immunofluorescence (×400) (*n* = 4 per group). *Notes*. (a) The white arrows indicate increased nuclear expression of NF-*κ*Bp65 in the DMAPA-Glyp+siRNA group; the asterisks indicate the weak nuclear signal of NF-*κ*Bp65 in the DMAPA-Glyp+NC group; the solid line rectangle indicates the magnifying region of the nuclear expression of NF-*κ*Bp65. (b) The fluorescence level counting of NF-*κ*Bp65 in the nuclei of the ciliary muscle was quantified to confirm NF-*κ*Bp65 nuclear translocation, and the values were represented in the bar graph. The error bars represent the standard deviation calculated from three parallel experiments. ^∗^*P* < 0.01, compared with the DMAPA-Glyp+NC group. Abbreviations: DAPI—4,6-diamidino-2-phenylindole; cb—ciliary body; cm—ciliary muscle; scle—sclera; DMAPA-Glyp—3-(dimethylamino)-1-propylamine-conjugated glycogen; NC—nonspecific control.

NF-*κ*Bp65 expression at the mRNA (24 h: *F* = 1.846, *df* = 35, *P* > 0.05; 48 h: *F* = 1.483, *df* = 35, *P* > 0.05; and 72 h: *F* = 0.943, *df* = 35, *P* > 0.05) and protein (24 h: *F* = 1.565, *df* = 35, *P* > 0.05; 48 h: *F* = 1.192, *df* = 35, *P* > 0.05; and 72 h: *F* = 2.033, *df* = 35, *P* > 0.05) levels showed no significant differences in the different groups at various time points after intracameral injection (Figure 5). Immunofluorescence staining of NF-*κ*Bp65 revealed increased nuclear expression of NF-*κ*Bp65 at 24, 48, and 72 h after transfection compared with the control group (24 h: *t* = −19.427, *df* = 6, *P* < 0.01; 48 h: *t* = −21.784, *df* = 6, *P* < 0.01; and 72 h: *t* = −18.228, *df* = 6, *P* < 0.01) (Figure 6).

### 3.6. Changes in MMP-2 Gene Expression after RNAi

**Figure 7:**
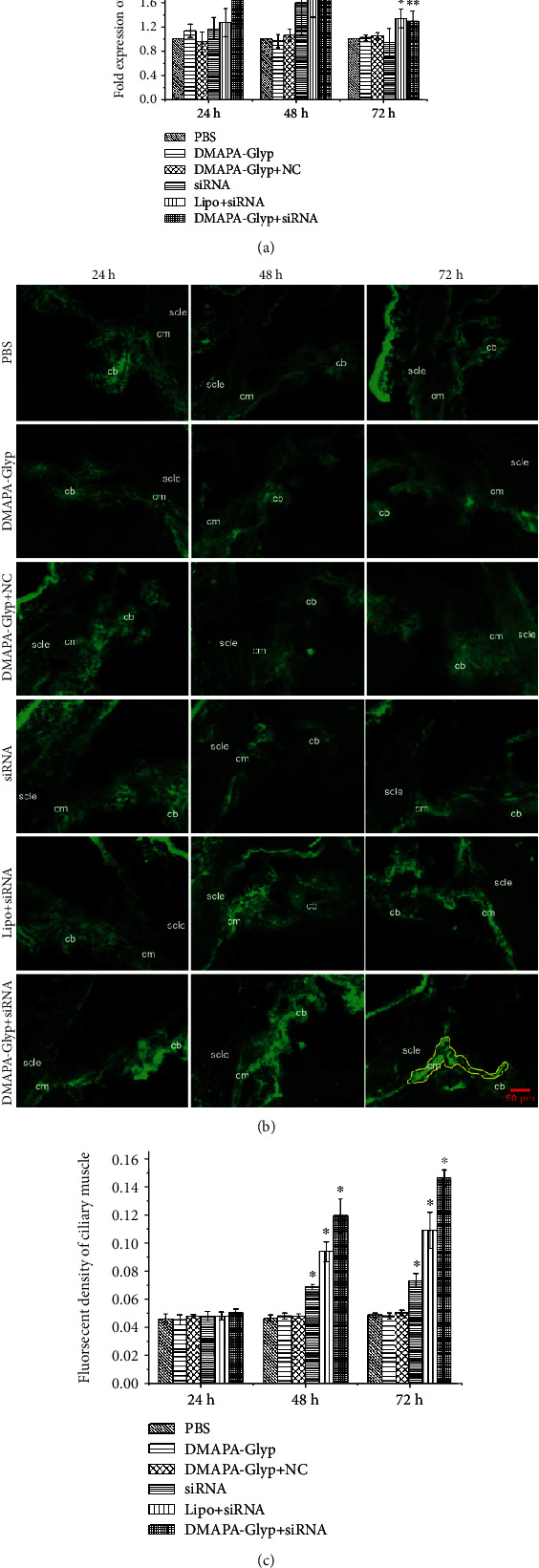
MMP-2 mRNA expression and activity at 24, 48, and 72 h after RNAi. *Notes*. (a) MMP-2 mRNA expression was quantified by real-time PCR. Expression levels were normalized with GAPDH. The error bars represent the standard deviation calculated from three parallel experiments (*n* = 6 per group). (b) The activity of MMP-2 was analyzed by *in situ* gelatin zymography. The stronger the green fluorescence in the ciliary muscle, the higher the MMP-2 activity (×200). The yellow solid line indicates the area of the ciliary muscle scanned for MMP-2 activity. (c) Fluorescence level counting in the area of the ciliary muscle was performed to measure the MMP-2 activity, and the values were represented in the bar graph. The error bars represent the standard deviation calculated from three parallel experiments (*n* = 4 per group). ^∗^*P* < 0.01 and ^∗∗^*P* < 0.05, compared with the PBS group, the DMAPA-Glyp group, and the DMAPA-Glyp+NC group. Abbreviations: PBS—phosphate-buffered saline; DMAPA-Glyp—3-(dimethylamino)-1-propylamine-conjugated glycogen; NC—nonspecific control; Lipo—Lipofectamine™ 2000; cb—ciliary body; cm—ciliary muscle; scle—sclera.

At 24 h after intracameral injection, MMP-2 mRNA expression significantly increased only in the DMAPA-Glyp+siRNA group compared with the three control groups (*F* = 11.448, *df* = 35, *P* < 0.01). At 48 h after transfection, MMP-2 mRNA expression significantly increased in the three I*κ*B*α*-siRNA-transfected groups. The largest increase, an increase of approximately 3-fold compared with the PBS group, occurred in the DMAPA-Glyp+siRNA group (*F* = 33.967, *df* = 35, *P* < 0.01). At 72 h after transfection, except for the siRNA group, the increase in MMP-2 mRNA expression in the DMAPA-Glyp+siRNA and Lipo+siRNA groups was statistically significant compared with the three control groups (*F* = 9.443, *df* = 35, *P* < 0.01, Figure 7(a)).

Changes in MMP-2 activity in the rat ciliary muscle after intracameral injection were examined using *in situ* gelatin zymography of MMP-2 in the frozen sections. The results showed that MMP-2 activity changed insignificantly in the three I*κ*B*α*-siRNA-transfected groups only at 24 h after transfection compared with the three control groups (*F* = 1.691, *df* = 23, *P* > 0.05). As the time of transfection was increased, the enzyme activity gradually increased (48 h: *F* = 100.540, *df* = 23, *P* < 0.01), reaching its highest level at 72 h (72 h: *F* = 167.557, *df* = 23, *P* < 0.01). Among the three I*κ*B*α*-siRNA-transfected groups, the DMAPA-Glyp+siRNA group showed the greatest elevation of MMP-2 activity. The MMP-2 activity increased to 2.5- and 3-fold at 48 and 72 h, respectively, compared with that in the PBS group (Figures 7(b) and 7(c)).

### 3.7. Changes in IOP in Rats after RNAi

**Figure 8:**
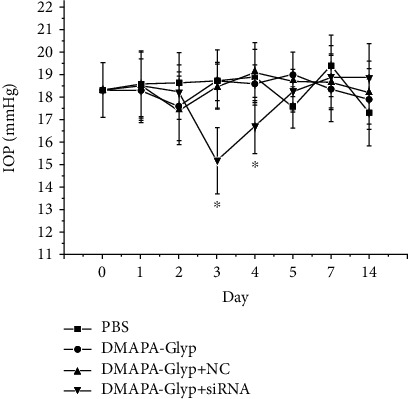
Changes in IOP in rats at 24, 48, and 72 h after RNAi. The error bars represent the standard deviation calculated from three parallel experiments (*n* = 10 per group). *Notes*. ^∗^*P* < 0.01, compared with the PBS group, the DMAPA-Glyp group, and the DMAPA-Glyp+NC group. Abbreviations: PBS—phosphate-buffered saline; DMAPA-Glyp—3-(dimethylamino)-1-propylamine-conjugated glycogen; NC—nonspecific control.

The baseline of the rat IOP was 18.33 ± 1.21 mmHg and remained at a relatively stable level. After RNAi, the IOP of the rats in the DMAPA-Glyp+siRNA group decreased to 15.20 ± 1.47 mmHg at 3 d and to 16.75 ± 1.24 mmHg at 4 d. These differences were statistically significant compared with the IOPs of the rats in the three control groups (3 d: *F* = 21.508, *df* = 39, *P* < 0.01; 4 d: *F* = 8.934, *df* = 39, *P* < 0.01). The IOP in the DMAPA-Glyp+siRNA group receded to the baseline at 5 d (Figure 8).

### 3.8. Evaluation of the Toxicity of the DMAPA-Glyp Derivative

**Figure 9:**
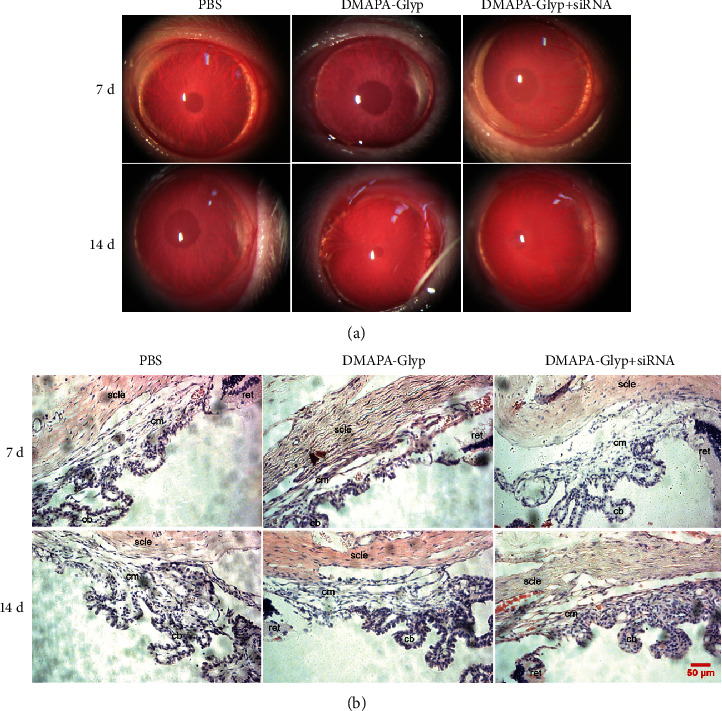
Evaluation of the toxicity of DMAPA-Glyp in a rat anterior segment. *Notes*. (a) Rat anterior segment photography. (b) HE staining of rat ciliary body (×200) (*n* = 5 per group). Abbreviations: cb—ciliary body; cm—ciliary muscle; ret—retina; scle—sclera; PBS—phosphate-buffered saline; DMAPA-Glyp—3-(dimethylamino)-1-propylamine-conjugated glycogen.

Anterior segment photography showed no occurrence of cataract, corneal edema, iris hyperemia, or hemorrhage in any of the groups at 7 and 14 d after intracameral injection (Figure 9(a)). HE staining revealed no significant inflammatory cell infiltration or pathological damage to the ciliary muscle or in the anterior chamber (Figure 9(b)).

## 4. Discussion

In this study, we prepared the DMAPA-Glyp/I*κ*B*α*-siRNA complex using the DMAPA-Glyp derivative as the vector. Such complex was transfected into rat ciliary muscle to explore the role of the I*κ*B/NF-*κ*B signaling pathway in the uveoscleral outflow pathway.

IOP was the key part of our study. Therefore, the effect of different anesthetics on intraocular pressure should be a concern. A study showed that chloral hydrate sedation for outpatient pediatric ophthalmic procedures had no impact on IOP [17]. Inhalational agents, such as desflurane, isoflurane, and sevoflurane, could decrease IOP by suppressing the diencephalon (experimental studies have shown that the diencephalon has a direct effect on IOP [18]), reducing aqueous humor production, increasing aqueous humor outflow, and relaxing the extraocular muscles [19]. It was generally believed that propofol induction caused a decrease in systemic arterial pressure, which might cause a sharp drop in IOP [20]. Many studies suggested that ketamine elevated IOP in pediatric patients [21] and in healthy dogs [22, 23]. By comparing the above agents, we finally chose chloral hydrate to avoid the effect on the results of IOP.

In the course of our experiment, intravitreal injection, ciliary muscle injection, or intracameral injection into a rat eye took only about 5 seconds, so that topical anesthesia with 0.5% proxymetacaine hydrochloride drops combined with chloral hydrate sedation was enough. Furthermore, our experiment was supervised by Prof. Yuqing Lan, and no rats died throughout the course of the experiment.

The siRNA-loaded cationic polymer complexes could effectively improve the pharmacokinetics and targeting of siRNA *in vivo* [24, 25]. Glycogen, coming from animals, is a naturally hyperbranched polysaccharide with no toxicity, good biocompatibility, and good biodegradability [26–29]. We have reported the synthesis method of the DMAPA-Glyp derivative, and the stable DMAPA-Glyp/siRNA complex could be prepared at their weight ratio of 5 [12]. Accordingly, the DMAPA-Glyp/siRNA complex was prepared based on such weight ratio here.

Selecting an effective delivery route for the safe and efficient delivery of siRNA to the target site is a prerequisite for successful *in vivo* RNAi. In the present study, we found that a more intense red fluorescence of Cy3-siRNA in the ciliary muscle was found at 24 h after intracameral injection than that was found after intravitreal or ciliary muscle injection. Moreover, the red fluorescence was located at the sites that displayed green fluorescence indicating immunofluorescence staining for *α*-SMA (smooth muscle *α*-actin), a specific component of smooth muscle, whose antibody exhibits no cross-reaction with desmin, nor does it react with other mesenchymal cells, epithelial cells, or cytoskeletal components. This suggests that the siRNA was effectively delivered to the ciliary muscle via intracameral injection which was selected as the delivery route for the subsequent experiments.

The DMAPA-Glyp/Cy3-siRNA complex was intracamerally injected into rat eyes to evaluate its transfection efficiency, using the classical cationic transfection reagent Lipofectamine™ 2000 loading Cy3-siRNA and naked Cy3-siRNA as the controls. We then observed the distribution of red fluorescence caused by Cy3-siRNA in the ciliary muscle of each group at 24, 48, and 72 h after transfection. Loading of the Cy3-siRNA onto DMAPA-Glyp resulted in a higher transfection efficiency with more red fluorescence in the ciliary muscle than the control groups. This may be related to the hyperbranched structure of DMAPA-Glyp which improves its gene transfection performance and the excellent protective effect of DMAPA-Glyp on siRNA [12]. In a study of the dynamics of aqueous humor and morphology in the uveoscleral pathway, Toris et al. [30] suggested that the difficulty of a given tracer in entering the uveoscleral pathway is closely related to its molecular weight. FITC-dextrans with molecular weights between 4000 and 150,000 rarely enter uveal vessels; instead, they mainly enter the suprachoroidal space through the ciliary muscle gap. Thus, it appears likely that the high transfection efficiency of DMAPA-Glyp is related to the molecular weight and the particle size of the DMAPA-Glyp/siRNA complex.

To evaluate the performance of DMAPA-Glyp as a vector for *in vivo* transfection, we used the Lipo/siRNA complex and naked siRNA as the controls and compared their inhibitory effects on I*κ*B*α* gene expression of the ciliary muscle after intracameral injection. The more remarkable gene silencing effect in the DMAPA-Glyp+siRNA group at the mRNA and protein levels indicates that the DMAPA-Glyp/siRNA complex shows significantly higher efficacy of suppression on I*κ*B*α* than the controls.

NF-*κ*B denotes a class of Rel protein dimer transcription factors that can specifically bind to DNA. The p50 and p65 heterodimers of NF-*κ*B have been intensively studied, as has their trimer with I*κ*B*α* [31]. In the resting state, the nuclear localization signal on p50 in the trimer is masked, and the trimer is retained in the cytoplasm and remains inactive. When cells are subjected to various extracellular stimuli, I*κ*B*α* is rapidly degraded, and the nuclear localization signal of NF-*κ*Bp50 is exposed; the p50 and p65 heterodimers then enter the nucleus and specifically bind to gene-specific sequences, thereby playing a regulatory role in transcription.

We suppressed I*κ*B*α* expression via RNAi in the rat ciliary muscle; we then observed the effects of this intervention on NF-*κ*Bp65 expression and nuclear translocation, as well as MMP-2 expression and activity. There were no significant changes in NF-*κ*Bp65 mRNA or protein expression at 24, 48, or 72 h after RNAi. However, immunofluorescence staining showed NF-*κ*Bp65 translocation into the nuclei after RNAi. This was consistent with our previous study in human ciliary muscle cells [9]. The anti-NF-*κ*Bp65 rabbit monoclonal antibody we used in this study could recognize endogenous levels of total NF-*κ*Bp65 protein (both the inactive form of the p65 subunit, bound to p50 and I*κ*B in the cytoplasm, and the active monomeric form in the nucleus). Therefore, despite the nuclear translocation of NF-*κ*Bp65, its total protein expression might not change significantly.

From real-time PCR, we found that all three I*κ*B*α*-siRNA-transfected groups showed significantly and maximally increased MMP-2 mRNA expression at 48 h after intracameral injection. The largest increase in MMP-2 mRNA expression occurred in the DMAPA-Glyp+siRNA group at all three time points. Substrate zymography is the method that is most commonly used to analyze the expression of MMPs. Here, we used *in situ* gelatin zymography to assay and localize MMP-2 activity in rat ciliary muscle. The results showed that the highest level of MMP-2 activity in the three I*κ*B*α*-siRNA-transfected groups was present at 72 h. The most obvious increase in MMP-2 activity occurred in the DMAPA-Glyp+siRNA group. These results indicate that along with NF-*κ*B activation and nuclear translocation, MMP-2 activity also increases in the rat ciliary muscle.

We measured the IOP of rats and determined whether the increase in MMP-2 activity in the rat ciliary muscle affects the IOP after RNAi. The handheld Tono-Pen tonometer is an electronic applanation tonometer [32]. The IOP value measured using the Tono-Pen is correlated with corneal thickness and ocular axial length [33], as well as the intensity and angle of the contact between the tonometer and the cornea. Because IOP fluctuates with circadian rhythm [34], we had the same experimenter measure the IOP of rats using the same positioning of the instrument at the same time of day for each measurement.

Whether the animal is anesthetized or not and the type of anesthetic used can affect the IOP. Jia et al. [35] reported that anesthetization decreases IOP and increases the difference in IOP between animals and that IOP measurement is more accurate when the animal is in an awake state. In the present study, the IOP of the rats was measured after topical anesthesia with 0.5% proxymetacaine hydrochloride drops. The baseline IOP measured in this way was 18.33 ± 1.21 mmHg. This is generally consistent with the mean normal IOPs in rats reported by Bakalash et al. [36, 37], i.e., 17.37 ± 2.19 mmHg and 19.41 ± 1.68 mmHg. However, Sawada and Neufeld [38], using a pneumotonometer, found the normal IOP of awake Wistar rats to be 11.6 ± 0.7 mmHg. Additionally, Wang et al. [39], using a Tono-Pen, found the normal IOP of awake Wistar rats to be 22.96 ± 0.18 mmHg. Our experimental results show varying differences from the IOP values reported in those studies. This may be related to the differences in the measuring instruments used, differences in the measurement time, the technique of the operator, and/or the compliance of the animals.

Husain et al. [40] found that Latanoprost increases the aqueous humor outflow in the uveoscleral pathway by activating MMPs in the ciliary body, thereby lowering the IOP of rats. In the present study, MMP-2 activity in the rat ciliary muscle significantly increased in the DMAPA-Glyp+siRNA group at 3 d after intracameral injection. And the IOP of the rats in this group decreased to the lowest level at the same time after RNAi. We conjecture that the IOP reduction after injection of the DMAPA-Glyp/siRNA complex may occur because activated MMP-2 following NF-*κ*B nuclear translocation degrades the ECM of the ciliary muscle and thus promotes uveoscleral outflow of the aqueous humor. The consequent change of the ECM in the ciliary muscle will be investigated in further studies. The IOP in the DMAPA-Glyp+siRNA group receded to the baseline at 5 d. The ocular hypotensive effect appeared to be short-lived in normotensive eyes of rats. Huang et al. [41] indicated that 0.5% timolol could lower the IOP in rat eyes with normal ocular pressure which was observed to last 6 hours after treatment, whereas 0.5% timolol still showed significantly great hypotension effects in a laser-induced ocular hypertension model in rats 7 days after treatment. Liu et al. [42] reported that RhoA siRNA (siRhoA) was applied to normal and DEX-induced elevated IOP mice by intracameral injection. In normal mice, injections of siRhoA induced decreases in IOP by 2 d, with recovery to baseline by 3 d, postinjection. For DEX-treated animals, IOP significantly decreased from 2 d to 5 d postinjection. The differences in IOP changes between the normal and hypertension model might be due to the different functional states of the aqueous humor outflow pathway. The hypotensive effect of DMAPA-Glyp/I*κ*B*α*-siRNA in the ocular hypertension rat model needs to be investigated in the future.

However, several studies indicated the opposite conclusion in that NF-*κ*B activity was a driver for increased outflow resistance in the TM. Hernandez et al. [43] showed that NF-*κ*B was necessary for TGF*β*2-induced ECM production and ocular hypertension. Wang et al. [44] showed that IL-1 produced endogenously by glaucomatous TM cells inhibited the apoptotic response to oxidative stress through NF-*κ*B and increased outflow facility perhaps through its ability to stimulate expression of MMPs. These studies focused on the conventional outflow pathway, and the changes in ciliary muscle cells governing the unconventional route need to be further explored.

A large amount of the DMAPA-Glyp/Cy3-siRNA complexes were found in the ciliary muscle after intracameral injection. Meanwhile, some of them were observed in the trabecular meshwork as well (Figures 1–3). In a review concerning unconventional aqueous humor outflow, Johnson et al. [45] mentioned in the direct measurement of its flow rate that outflow of the tracer introduced into the anterior chamber through the conventional pathway is relatively fast (a minute or less) and fairly insensitive to tracer molecular size. In contrast, tracers draining through the unconventional pathway are retarded or captured as they move through the unconventional outflow pathway such that their transit may take up to two hours depending on tracer size, animal species, and dimensions of the eye. Thus, we have reason to infer that the longer time for which the DMAPA-Glyp/Cy3-siRNA complexes stay in the unconventional outflow pathway provides the complexes more opportunities to transfect into the ciliary muscle. Additionally, the appropriate molecular weight and particle size of the DMAPA-Glyp/siRNA complex, as mentioned above, induce the high transfection efficiency in the ciliary muscle. Therefore, we consider that the unconventional outflow pathway is the major contributor to the effect on IOP in response to I*κ*B*α* gene silencing. As for the role of the DMAPA-Glyp/I*κ*B*α*-siRNA complexes in the conventional outflow pathway, further studies will be conducted.

In addition to efficacy, a desired vector must meet the requirement of safety. In our previous study [12], the DMAPA-Glyp and DMAPA-Glyp/siRNA complex showed significantly lower cytotoxicity against human retinal pigment epithelial (hRPE) cells when compared to branched polyethylenimine (bPEI). In this study, no pathological damage to the ciliary muscle or the anterior chamber was found in rats injected with DMAPA-Glyp alone or with the DMAPA-Glyp/siRNA complex. No rats died after injection with those above throughout the course of the experiment. These results prove that DMAPA-Glyp has excellent biocompatibility and no toxicity in rats.

## 5. Conclusions

Downregulation of I*κ*B*α* expression in the ciliary muscle plays a crucial role in reducing the IOP values of rats. I*κ*B*α* may become a new molecular target for lowering IOP in glaucoma. The DMAPA-Glyp derivative is safe and feasible as an effective siRNA vector in rat eyes.

## Figures and Tables

**Table 1:** Nucleotide sequences of primers for PCR.

Gene	Primer sequence (5′-3′)		Product size (bp)
I*κ*B*α*	Forward	TGACCATGGAAGTGATTGGTCAG	95
	Reverse	GATCACAGCCAAGTGGAGTGGA	
NF-*κ*Bp65	Forward	CGACGTATTGCTGTGCCTTC	139
	Reverse	TTGAGATCTGCCCAGGTGGTA	
MMP-2	Forward	TGTGGCACCACCGAGGATTA	85
	Reverse	CTGAATTTCCACCCACAGTGGAC	
GAPDH	Forward	GGCACAGTCAAGGCTGAGAATG	143
	Reverse	ATGGTGGTGAAGACGCCAGTA	

Abbreviations: MMP-2—matrix metalloproteinase-2; GAPDH—glyceraldehyde 3-phosphate dehydrogenase.

**Table 2:** Grouping and treatment of animals.

Group	Treatment^a^
PBS group	PBS
DMAPA-Glyp group	DMAPA-Glyp solution containing 25 *μ*g of DMAPA-Glyp
DMAPA-Glyp+NC group	DMAPA-Glyp and NC-siRNA complex containing 25 *μ*g of DMAPA-Glyp and 5 *μ*g of NC-siRNA
siRNA group	I*κ*B*α*-siRNA solution containing 5 *μ*g of I*κ*B*α*-siRNA
Lipo+siRNA group	Lipofectamine™ 2000 and I*κ*B*α*-siRNA complex containing 1.5 *μ*l of Lipofectamine™ 2000 and 5 *μ*g of I*κ*B*α*-siRNA
DMAPA-Glyp+siRNA group	DMAPA-Glyp and I*κ*B*α*-siRNA complex containing 25 *μ*g of DMAPA-Glyp and 5 *μ*g of I*κ*B*α*-siRNA

^a^Intracameral injection with a volume of 10 *μ*l per eye. Abbreviations: PBS—phosphate-buffered saline; DMAPA-Glyp—3-(dimethylamino)-1-propylamine-conjugated glycogen; NC-siRNA—nonspecific control siRNA; Lipo—Lipofectamine™ 2000.

## Data Availability

The data used to support the findings of this study are included within this article.
